# Neurophysiological assessment of spinal cord injuries in dogs using somatosensory and motor evoked potentials

**DOI:** 10.1186/s13028-017-0305-0

**Published:** 2017-06-12

**Authors:** Maria Claudia Campos Mello Inglez de Souza, Ricardo José Rodriguez Ferreira, Geni Cristina Fonseca Patricio, Julia Maria Matera

**Affiliations:** 1 Department of Surgery, School of Veterinary Medicine and Animal Science, University of São Paulo, Cidade Universitária, Prof. Dr. Orlando Marques de Paiva, 87, São Paulo, SP 05508-270 Brazil; 2 Orthopedic and Traumatology Institute, School of Medicine, University of São Paulo, Rua Dr. Ovídio Pires de Campos, 333, São Paulo, SP 05403-010 Brazil

**Keywords:** Neurophysiology, Transcranial electrical stimulation, SSEPs, MEPs

## Abstract

Somatosensory evoked potentials (SSEPs) and motor evoked potentials (MEPs) are non-invasive neurophysiological tests that reflect the functional integrity of sensory and motor pathways. Despite their extensive use and description in human medicine, reports in veterinary medicine are scarce. SSEPs are obtained via peripheral stimulation of sensory or mixed nerves; stimulation induces spinal and cortical responses, which are recorded when sensory pathways integrity is preserved. MEPs can be obtained via transcranial electrical or magnetic stimulation; in this case, thoracic and pelvic limb muscle responses are captured if motor pathways are preserved. This review describes principles, methodology and clinical applicability of SSEPs and MEPs in companion animal medicine. Potential interferences of anesthesia with SSEP and MEP recording are also discussed.

## Background

Spinal cord injuries may lead to clinical signs ranging from localized pain to paresis or plegia, with or without preservation of conscious pain perception. Along with regenerative therapy, more attention should be given to diagnostic modalities and objective tools designed to monitor neural pathways as a predictor of outcome in veterinary medicine.

Neurophysiological studies involve a range of tests designed to record action potentials along sensory and motor pathways; such tests provide data on injury site and severity and help to assess the progression of spinal cord lesions (i.e., improvement or deterioration) [1, 2]. Some modalities can specifically evaluate the peripheral nervous system, as nerve conduction studies, others will also evaluate the central nervous system, as somatosensory evoked potentials and motor evoked potentials, as described in this review. Intraoperative monitoring during spinal surgery is also possible, with improved prevention of iatrogenic injuries and more accurate prognosis [1, 3]. This review describes basic principles, methodology and clinical applicability of evoked potential tests in small animals.

## Search strategy

This overview was provided through searches of Pubmed (http://www.ncbi.nlm.nih.gov/pubmed) and Google Scholar (https://scholar.google.com), using the terms “motor evoked potentials, somatosensory evoked potentials, SSEPs and MEPs”. The titles and abstracts were evaluated and pertinent articles related to the current study were identified. Full-text manuscripts were then collected and assessed in detail. Personal archives were used to illustrate SSEPs and MEPs recordings.

## Review

Evoked potentials are neurophysiological tests designed to assess, among others, the functionality of neural pathways involved in spinal cord injuries. Somatosensory evoked potentials (SSEPs) correspond to spinal and cortical sensory responses recorded following electrical stimulation of a peripheral nerve, and reflect the functionality of ascending sensory pathways. In contrast, motor evoked potentials (MEPs) correspond to peripheral muscular responses recorded following motor cortex stimulation and reflect the functionality of descending motor pathways [2, 4].

Neurophysiological techniques are extremely popular in human medicine and are particularly indicated for intraoperative monitoring during spinal surgery; however, reports on neurophysiological assessment of spinal cord injuries in veterinary medicine are scarce. Neurophysiological tests have been employed to record SSEPs [5, 6] and MEPs in healthy animals [7–10] and in cases of cervical spondylomyelopathy [11, 12], intervertebral disk disease [13], lumbosacral stenosis [14], hereditary diseases [15, 16] and traumatic injuries [17]. Their use in evaluation of responses to regenerative therapy [18] and in intraoperative monitoring during cervical spinal surgery [19] has also been reported.

### Somatosensory evoked potentials (SSEPs)

#### Principles

Somatosensory evoked potentials reflect the integrity of large-diameter sensory nerve fibers running through the dorsal funiculus. Potentials generated via stimulation of peripheral nerves are recorded at different levels of the nervous system, such as peripheral nerve, plexus, nerve roots, spinal cord segments and the sensory cortex. The tibial and median nerves are the nerves of choice for stimulation in pelvic and thoracic limbs respectively [2, 5, 6]. Somatosensory evoked potentials correspond to the conscious proprioception pathway; therefore, SSEPs do not represent smaller diameter fibers conveying pain and temperature sensation and may be altered or absent even in individuals with nociception [2].

#### Methodology

Stainless steel electrode pairs (i.e., cathode and anode) inserted into the subcutaneous tissue over the distal end of the target nerve are used for stimulation. Proximal stimulation can recruit more fibers and then promote larger potentials, but can also cause more muscle reflex activity. Stimulation just above the carpus for the median nerve and just above the tarsus for the tibial nerve can stimulate enough number of fibers avoiding excessive muscle activity [2, 20]. The most negative electrode (cathode) causes axon membrane depolarization, and should be placed 2 cm proximal to the anode, in order to avoid the anodal block of conduction [5, 6]. Surface electrodes are commonly used in humans; however, monopolar needle electrodes are recommended in dogs and cats due to greater skin thickness, larger amounts of subcutaneous fat and the presence of fur [2, 5]. Continuous stimuli applied to one limb at a time help to assess lesion lateralization. Pulse duration of 0.1 ms is most widely used. Stimulus intensity is related to recruitment of nerve fibers and the larger is the diameter of a fiber nerve, more easily it is stimulated. Moreover, with increasing stimulus intensity, more nerve fibers are recruited [20]. Therefore, pulse intensity (mA) is adjusted until a barely visible distal limb contraction is elicited indicating proper stimulation of motor fibers in mixed nerves such as the tibial and median nerves, and this is subject to individual variation. Once a good motor twitch is obtained, increasing the stimulus intensity further does not increase SSEPs amplitudes [20, 21].

Stimulus rates usually range from 2 to 5 Hz [4–6, 13], as amplitudes of scalp-recorded SSEPs can be modified by pulse rates above 2 Hz and spine recorded SSEPs by rates above 4 Hz [20]. Electrodes connected to the stimulator cause axons depolarization, with centrally (also peripherally) propagation, and SSEPs can be recorded from the spinal cord ascending tracts and finally in the scalp.

Filters are used to avoid activity not related to the generator under study that would interfere with the recordings. Commonly, a window from 10 to 4000 Hz in spine recorded SSEP and 10–3000 Hz for the scalp-recorded SSEP is used [5, 6, 17]. As signals under study are of very small magnitude, “Signal averaging” is applied to differentiate signals of interest from other interferences. This signal is time-locked to the stimulus, while noise is a random event. With averaging of repeated responses, noise is averaged out and the signal is averaged in [20].

As mentioned before, the sensory pathway may be further subdivided and topographed using electrode pairs placed at different spinal segments [5, 14, 17]. For spinal cord SSEPs, the active recording electrodes are placed in the dorsal midline parallel to the edges of adjacent to spinous process, aiming the center of the interarcuate ligament. The reference electrode is positioned in the paraspinal muscles 1–3 cm lateral to the recording electrode, at the same level. The active recording electrode can be advanced cranially to study different spinal cord segments, and also adjacent to a lesion, so that is possible to delimit the injury site. Ground electrode can be inserted subcutaneously preferably over a bone prominence, as these are electrically inactive regions [5, 13, 14, 17]. Along the spine, four contributions to SSEP recording can be identified. They are the root component (at L7–S1 level), the cord dorsum potential (in the caudal lumbar area), the ascending evoked potential (at more rostral levels) and the medullary component (at the level of the cisterna magna) [5, 20].

The root component is a potential that originates in the cauda equina nerve roots and can be detected on the three most caudal intervertebral spaces.

The cord dorsum potential is a triphasic potential that originates in the region of the spinal cord segments that receive input from sensory and mixed peripheral nerves. Therefore, it assess integrity of proximal sensory nerves, dorsal nerve roots, and spinal cord dorsal horn gray matter [22].

The ascending evoked potential is a compound action potential of small amplitude that can be difficult to detect in cranial thoracic and cervical areas due to the difficulty of inserting the electrodes properly. Finally, the medullary component possibly originates in the cervical and medullary nuclei [20].

Another feature of spine-recorded SSEP is the evoked injury potential, which is the spinal evoked response obtained by volume recording after injury to the cord, and represent the action potentials ascending the cord tracts up to the site of injury but not passing it. It has also been used as an intraoperative localizing tool for acute spinal cord injury [20, 23].

For scalp recording, the active (also called recording or different) electrode can be placed in the central zone, subcutaneously [5, 13, 17, 19]. Corkscrew (spiral) needle electrodes can be used for better adherence and minimization of external interferences [2, 5]. In the scalp, electrode position reflects the somatosensory area. A reference electrode placed centrally/frontally on the median plane and a ground electrode in the neck or on the forelimb position are also employed [2, 5].

Electrode pairs are connected to preamplifiers, then to specific channels in the equipment [4, 5]. Graphical representations of digitally recorded latency and amplitude data are used to investigate conduction disturbances along the sensory spinal tract [2, 4, 5].

#### Recordings

**Fig. 1:**
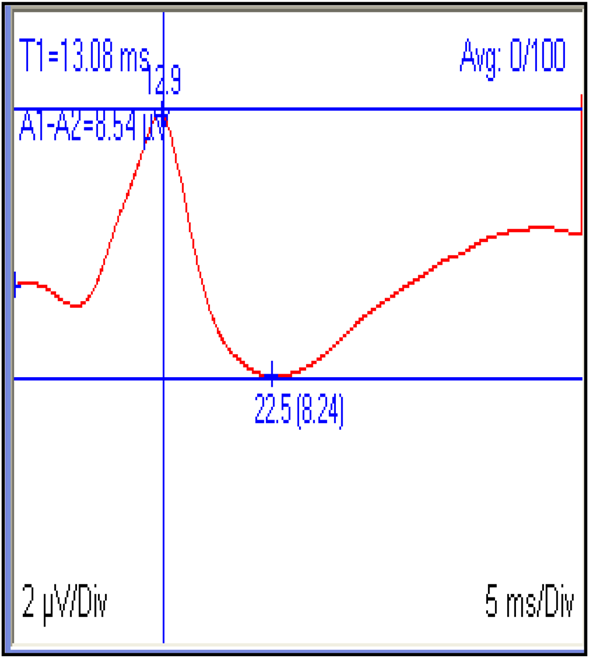
Somatosensory evoked potentials (SSEPs) recorded from scalp following median nerve stimulation in a dog. Latency (T1 = 13.08 ms; *vertical bar*) and peak-to-peak amplitude (A1–A2 = 8.54 µV; *horizontal bars*) measurements are displayed. Gain = 2 µV/div; sweep speed = 5 ms/div

Somatosensory evoked potential recordings are displayed on a computer screen; recordings and waves corresponding to each limb are shown in different windows, which can be adjusted for improved visualization. Figure 1 shows an example of SSEPs recorded from thoracic limb in a dog. Manually managed cursors are used to measure wave amplitude and latency [2, 4–6].

#### Clinical applicability

**Fig. 2:**
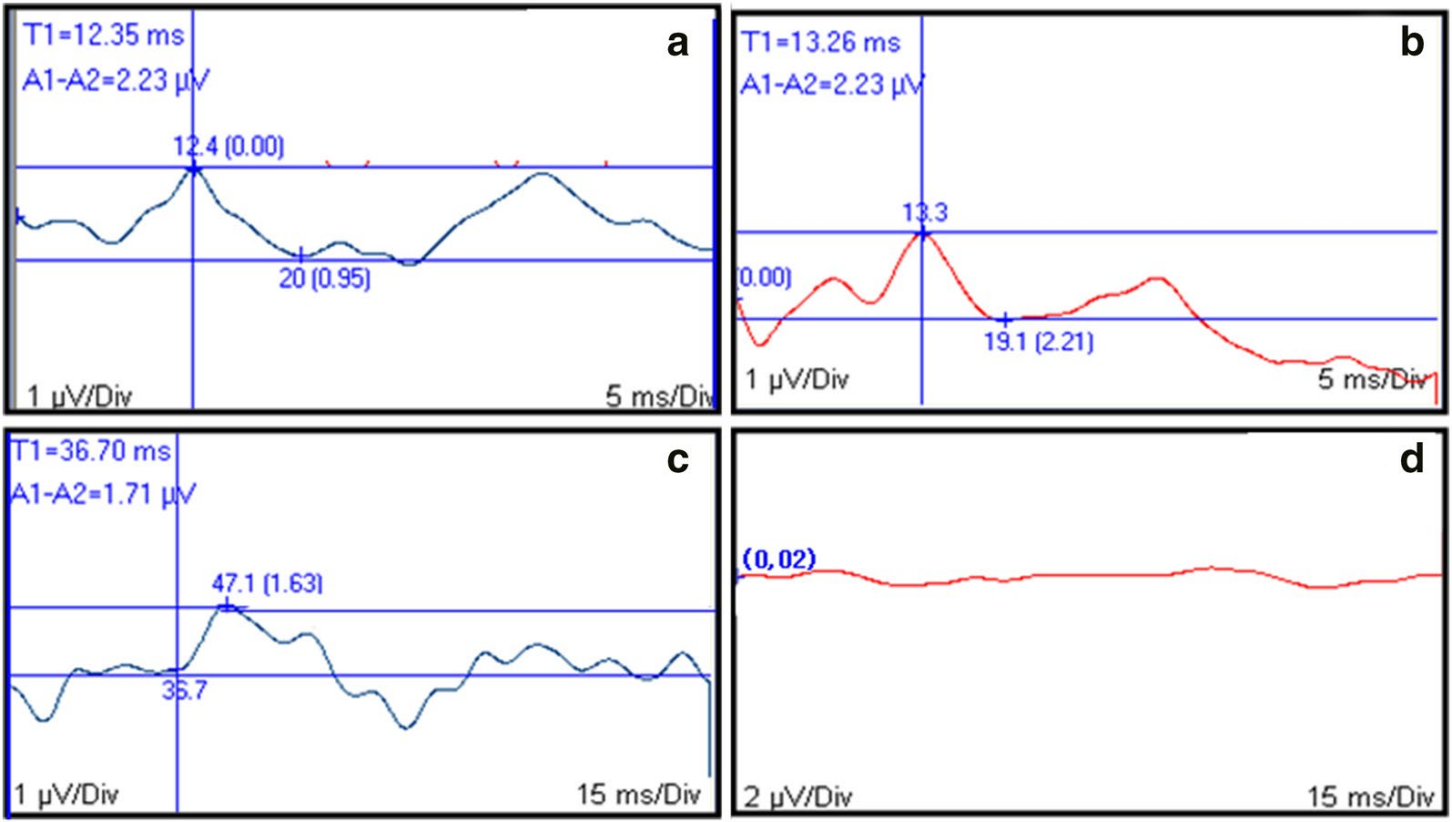
Somatosensory evoked potentials (SSEPs) recorded from a dog presenting with thoracolumbar spinal injury. Tracings obtained following left (**a**) and right (**b**) median nerve and left (**c**) and right (**d**) tibial nerve stimulation. Lack of SSEP following right pelvic limb stimulation reflects a spinal lesion caudal to right-sided cervical enlargement. Latency (ms; *vertical bar*) and peak-to-peak amplitude (µV) measurements are displayed. Gain = 1 µV/div (**a**, **b** and **c**) or 2 µV/div (**d**); sweep speed = 5 ms/div (**a**, **b**) or 15 ms/div (**c**, **d**)

Somatosensory evoked potentials reflect sensory pathway conductivity and integrity in areas that cannot be accessed using other electrodiagnostic tests and may therefore be used for investigation of central and peripheral neurological conditions [5]. The method is thought to be sensitive and specific for detection of peroperative complications, is user-friendly and can be combined with other neurophysiological diagnostic modalities [24, 25]. Thoracolumbar spinal cord injuries are expected to generate normal thoracic limb recordings and deteriorated pelvic limb recordings, with the magnitude of changes reflecting injury severity (i.e., the more severe the injury, the longer the latency and the lower the amplitude); alternatively, SSEPs may not be recorded [26]. In lateralized lesions such as lateral intervertebral disk extrusion, evidence of lateralization should be apparent in recordings (Fig. 2). In human medicine, these modalities include monitoring many neurosurgical or orthopedic spine surgeries, such as embolization or tumor resections, aneurysm repairs, peripheral nervous system surgeries, thus helping the safe surgical approach [2].

#### Observations

Somatosensory evoked potentials may be influenced by mechanical factors, ischemic conditions, systemic hypotension, hypothermia, injectable anesthetics such as thiopental sodium, pentobarbital and ketamine hydrochloride, and volatile agents such as isoflurane, sevoflurane and nitric oxide [2, 24]. Latency changes >10% and drops in amplitude of more 50% are alarming signs in intraoperative monitoring of human patients and should be reported to the surgeon in charge [27]. Somatosensory evoked potentials reflect the functional integrity of the dorsal columns and therefore do not represent motor pathways, which may be individually compromised [2, 24, 27].

### Motor evoked potentials (MEPs)

#### Principles

Motor evoked potentials assess motor pathway integrity from the cerebral cortex to the muscles and can be generated via transcranial magnetic [8, 10–12] or electrical stimulation [3, 7, 28]. While electrical stimulation is based on direct stimulation via subcutaneous electrodes inserted into the scalp, in transcranial magnetic stimulation a coil is used to generate magnetic fields that are then converted into electric potentials. Both methods induce depolarization and trigger action potentials that propagate along descending pathways related to the pyramidal and extrapyramidal systems; the first fibers descending from the cortex to the spinal cord form the corticospinal system [2, 10]. Extrapyramidal pathways correspond primarily to the rubrospinal, reticulospinal, tectospinal and vestibulospinal tracts and are particularly relevant in dogs [29]. The need for anesthesia is a major downside of transcranial electrical stimulation, as muscle contractions are painful, besides anticipated pain can occur [7].

In contrast, transcranial magnetic stimulation can be performed in sedated animals [8, 12] and does not require special preparation in humans [10]. However, electrical stimulation is less impacted by anesthetic drugs and is therefore the method of choice for intraoperative monitoring [2]. Also, motor responses induced via transcranial magnetic stimulation are easier to capture following voluntary movements of the target limb made upon request, which is not applicable to animal patients [2].

#### Methodology

For magnetic stimulation, a coil of wire generates the magnetic field, and it is positioned over the motor cortex, with the purpose to create a pulsed electric current [10]. For electric stimulation, cork screw electrodes provide better attachment to the scalp for proper transcranial stimulation and should be inserted centrally and above the left and right hemispheres, then connected to the stimulator. Active electrodes connected to the anode are expected to elicit better responses on the contralateral side. Multi pulses of 0.05 ms duration, individually adjusted to supramaximal intensity and frequency of 250 Hz are used [2]. Potentials are captured via needle electrodes inserted into target muscles, particularly those caudal to the injury site, although cranially located muscles may be employed as sentinels. The extensor carpi radialis and cranial tibial muscles (thoracic and pelvic limbs respectively) are the muscles of choice for MEP capture in dogs [11, 12, 18].

#### Recordings

**Fig. 3:**
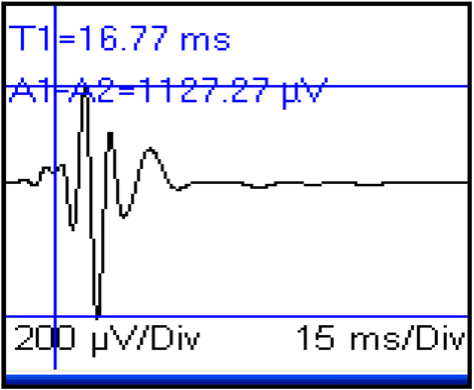
Motor evoked potentials recorded from the extensor carpi radialis muscle following transcranial electrical stimulation in a dog. Latency (T1 = 16.77 ms; *vertical bar*) and peak-to-peak amplitude (A1–A2 = 1127.27 µV; *horizontal bars*) measurements are displayed. Gain = 200 µV/div; sweep speed = 15 ms/div

As with SSEPs, MEP wave latency and amplitude values are displayed and measured on a computer screen (Fig. 3); SSEPs and MEPs should not be recorded simultaneously due to potential stimulation artifact interferences [4].

#### Clinical applicability

**Fig. 4:**
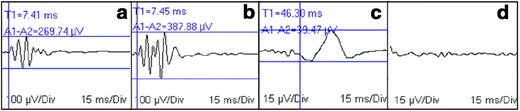
Motor evoked potential recordings (MEPs) in a dog presenting with thoracolumbar spinal injury. Tracings obtained following left (**a**) and right (**b**) extensor carpi radialis and left (**c**) and right (**d**) cranial tibial muscle stimulation. MEP capture in **a**, **b** and **c** and absence in **d** is consistent with right-sided spinal lesion. Latency (ms; *vertical bar*) and peak-to-peak amplitude (µV) measurements are displayed. Gain = 100 µV/div (**a**, **b**) or 15 µV/div (**c**, **d**); sweep speed = 15 ms/div

Motor evoked potentials have similar clinical applicability to SSEPs; however, motor pathways are reflected instead, and therefore a different system with specific functions and anatomical location [2]. Normal thoracic limb MEPs with altered pelvic limb MEP latency and amplitude should be expected in thoracolumbar spinal injuries. As with SSEPs, recording abnormalities are consistent with lesion severity [30, 31], (i.e., the more severe the injury, the grater the latency and the lower the amplitude), although they do not seem to correlate with prognosis for recovery. MEPs may not be recorded caudal to the injury site [32, 33] (Fig. 4).

#### Observations

Motor evoked potential changes occur more rapidly than SSEP changes under ischemic conditions; therefore this technique is more sensitive for detection of intraoperative spinal injuries [4, 24, 32, 33].

### Anesthesia in SSEP and MEP assessment

Chemical restriction is required for SSEP and MEP recording in veterinary medicine; however, common sedating and anesthetic agents may attenuate or even suppress motor and somatosensory responses. Hypnotic-opioid combinations such as intravenous propofol and remifentanil are routinely used in human patients [25, 28, 34] submitted to electrical stimulation. The hyperpolarization (i.e., creation of a more negative resting potential in cell membranes) induced by volatile anesthetic agents such isoflurane decreases neuronal excitability and may prevent action potentials from reaching motor cortex and motor neuron depolarization thresholds [27, 34–36]. Several anesthetic protocols have been described in veterinary medicine. The use of drugs such as xylazine and dexamedetomidine, midazolam, sufentanil [5, 12, 37–39], ketamine, methohexital and isoflurane [37, 40] has been reported; anesthetic induction and maintenance through constant rate infusion of propofol [15, 35] has also been proposed. While MEPs can be obtained via transcranial magnetic stimulation in sedated patients, strong contraction of masticatory muscles induced by electrical stimulation dictates the need for general anesthesia and the use of protectors to prevent tongue laceration when this technique is employed. Body temperature oscillations may interfere with recordings; therefore, this parameter must be monitored [10, 19].

#### SSEPs and MEPs in veterinary medicine

Application of these diagnostic modalities in veterinary medicine is still limited, and two major limiting factors should be mentioned. First, different anesthetic protocols, mainly with substances that strongly suppress cortical activity, can definitely impair proper recordings. Second, mild clinical signs due to spinal cord injury can drastically influence, or even prevent recordings. More investigation and standardization should be determinant for better and more trustable results, and more information extracted from human medicine should be of great benefit to application in animals. SSEPs and MEPs have been used alone or in combination to complement neurological examination, as well as for disease characterization and functional classification of spinal cord injuries [15–17, 20, 26, 31, 37, 40–42]. Several aspects need to be further investigated and defined before results can be compared between studies. Electrode insertion sites, particularly of scalp electrodes used for SSEP capture or MEP induction via transcranial magnetic or electric stimulation, must be standardized. In dogs, extensive anatomical variability in head shape (e.g., brachycephalic vs. dolichocephalic breeds) is likely to interfere with correct location of stimulation and signal capture sites. Latency and amplitude reference values are also open to question, given the wide variability even between individuals of the same breed [11, 31]. A single study describes the use of SSEPs for intraoperative monitoring of dogs with spinal cord dysfunction [19]. The value of evoked potentials possibly resides in the contribution of such tests to objective assessment of recovery or deterioration of neurological conditions via paired or serial comparisons of recordings obtained from the same animal. Also, intraoperative monitoring significantly increases procedure safety (i.e., prevention of iatrogenic lesions) and prognostication accuracy. Just as human patients, dogs and cats would certainly benefit from intraoperative neurophysiological monitoring during spinal surgery [43, 44].

## Conclusions

Somatosensory and motor evoked potentials reflect the functional integrity of ascending (sensory) and descending (motor) pathways and therefore have diagnostic and prognostic value. Somatosensory and motor evoked potentials constitute promising tools for assessment and follow up of neurological conditions and provide significant contributions to intraoperative monitoring of spinal procedures.

## References

[1] Langeloo DD, Journée HL, De Kleuver M, Grotenhuis JA (2000). Criteria for transcranial electrical motor evoked potential monitoring during spinal deformity surgery: a review and discussion of the literature. Neurophysiol Clin.

[2] Simon MV. Intraoperative neurophysiology: a comprehensive guide to monitoring and mapping. Ed. New York: Demos Medical; 2009.

[3] Szelényi A, Kothbauer KF, Deletis V (2007). Transcranial electric stimulation for intraoperative motor evoked potential monitoring: stimulation parameters and electrode montages. Clin Neurophysiol.

[4] Ferreira RJR. Monitoramento neurofisiológico intraoperatório nas cirurgias espinais. In: Tratado de medicina e reabilitação. Rio de Janeiro: Guanabara Koogan; 2010. p. 24–39.

[5] Pellegrino FC, Sica REP (2005). Potenciales evocados somatosensitivos (PESS) obtenidos por estimulación del nervio mediano (registros espinal y craneano) en caninos. InVet.

[6] Vanderzant CW, Schott RJ, Natale JE, Pondo CA, D’alecy LG (1989). Somatosensory evoked potentials of the dog: recording techniques and normal values. J Neurosci Methods.

[7] Strain GM, Prescott-Mathews JS, Tedford BL (1990). Motor potentials evoked by transcranial stimulation of the canine motor cortex. PVN.

[8] Amendt HL, Siedenburg JS, Steffensen N, Söbbeler FJ, Schütter A, Tünsmeyer J (2016). Transcranial magnetic stimulation with acepromazine or dexmedetomidine in combination with levomethadone/fenpipramide in healthy beagle dogs. Vet J.

[9] Ferreira R, Oliveira AR, Barros Filho TEP (2005). Padronização da técnica para captação do potencial evocado motor em ratos através da estimulação elétrica transcraniana. Acta Ortop Bras.

[10] Nollet H, Van Ham L, Deprez P, Vanderstraeten G (2003). Transcranial magnetic stimulation: review of the technique, basic principles and applications. Vet J.

[11] Da Costa RC, Poma R, Parent JM, Partlow G, Monteith G (2006). Correlation of motor evoked potentials with magnetic resonance imaging and neurologic findings in Doberman Pinschers with and without signs of cervical spondylomyelopathy. Am J Vet Res.

[12] Martin-Vaquero P, Da Costa RC (2014). Transcranial magnetic motor evoked potentials in Great Danes with and without clinical signs of cervical spondylomyelopathy: association with neurological findings and magnetic resonance imaging. Vet J.

[13] Poncelet L, Michaux C, Balligand M (1993). Somatosensory potentials in dogs with naturally acquired thoracolumbar spinal cord disease. Am J Vet Res.

[14] Meij BP, Suwankong N, Van Den Brom WE, Venker-Van Haagen AJ, Hazewinkel HA (2006). Tibial nerve somatosensory evoked potentials in dogs with degenerative lumbosacral stenosis. Vet Surg.

[15] Vanhaesebrouck AE, Van Soens I, Poncelet L, Duchateau L, Bhatti S, Polis I (2010). Clinical and electrophysiological characterization of myokymia and neuromyotonia in Jack Russell Terriers. J Vet Intern Med.

[16] Harcourt-Brown TR, Belshaw Z, Parker JE, Jeffery ND, Granger N (2011). Effects of syringomyelia on electrodiagnostic test results in Cavalier King Charles Spaniels. Am J Vet Res.

[17] Şenel OO, Şirin YS, Önyay T, Beşalti O (2012). Evaluation of spinal somatosensory evoked potentials in cats with traumatic spinal cord injury without deep pain perception. Ank Üniv Vet Fak Derg.

[18] Granger N, Blamires H, Franklin RJ, Jeffery ND (2012). Autologous olfactory mucosal cell transplants in clinical spinal cord injury: a randomized double-blinded trial in a canine translational model. Brain.

[19] Okuno S, Nakamura A, Kobayashi T, Orito K (2005). Effectiveness of intraoperative somatosensory evoked potential monitoring during cervical spinal operations on animals with spinal cord dysfunction. J Vet Med Sci.

[20] Poncelet L (1999). Electrophysiological assessment of spinal cord function through somatosensory evoked potentials in dogs. Vet Neurol Neurosurg J.

[21] Cozzi P, Poncelet L, Michaux C, Balligand M (1998). Effect of stimulus intensity on spinal cord somatosensory evoked potential in dogs. Am J Vet Res.

[22] Cuddon PA, Delauche AJ, Hutchison JM (1999). Assessment of dorsal nerve root and spinal cord dorsal horn function in clinically normal dogs by determination of cord dorsum potentials. Am J Vet Res.

[23] Schramm J, Krause R, Shigeno T, Brock M (1983). Experimental investigation on the spinal cord evoked injury potential. J Neurosurg.

[24] Pelosi L, Lamb J, Grevitt M, Mehdian SM, Webb JK, Blumhardt LD (2002). Combined monitoring of motor and somatosensory evoked potentials in orthopaedic spinal surgery. Clin Europhysiol.

[25] Costa P, Bruno A, Bonzanino M, Massaro F, Caruso L, Vincenzo I (2007). Somatosensory and motor evoked potential monitoring during spine and spinal cord surgery. Spinal Cord.

[26] Shores A, Redding RW, Knecht CD (1998). Spinal evoked potentials in dogs with acute compressive thoracolumbar spinal cord disease. Am J Vet Res.

[27] Gavaret M, Jouve JL, Péréon Y, Accadbled F, André-Obadia N, Azabou E (2013). Intraoperative neurophysiologic monitoring in spine surgery. Developments and state of the art in France in 2011. Orthop Traumatol Surg Res.

[28] Tsutsui S, Yamada H, Hashizume H, Minamide A, Nakagawa Y, Iwasaki H (2013). Quantification of the proportion of motor neurons recruited by transcranial electrical stimulation during intraoperative motor evoked potential monitoring. J Clin Monit Comput.

[29] Lorenz MD, Coates JR, Kent M (2011). Handbook of veterinary neurology.

[30] Kanchiku T, Taguchi T, Kaneko K, Fuchigami Y, Yonemura H, Kawai S (2001). A correlation between magnetic resonance imaging and electrophysiological findings in cervical spondylotic myelopathy. Spine.

[31] Sylvestre AM, Cockshutt JR, Parent JM, Brooke JD, Holmberg DL, Partlow GD (1993). Magnetic motor evoked potentials for assessing spinal cord integrity in dogs with intervertebral disc disease. Vet Surg.

[32] Owen J, Laschinger J, Bridwell K, Shimon S, Nielsen C, Dunlap J (1988). Sensitivity and specificity of somatosensory and neurogenic-motor evoked potentials in animals and humans. Spine.

[33] Hilibrand AS, Schwartz DM, Sethuraman V, Vaccaro AR, Albert TJ (2004). Comparison of transcranial electric motor and somatosensory evoked potential monitoring during cervical spine surgery. J Bone Joint Surg Am.

[34] Tamkus AA, Rice KS, Kim HL (2014). Differential rates of false-positive findings in transcranial electric motor evoked potential monitoring when using inhalational anesthesia versus total intravenous anesthesia during spine surgeries. Spine J.

[35] Van Soens I, Struys MM, Polis IE, Tshamala M, Nollet H, Bhatti SF (2009). Effects of sedative and hypnotic drug combinations on transcranial magnetic motor evoked potential, bispectral index and ARX-derived auditory evoked potential index in dogs. Vet J.

[36] Kalkman CJ (1997). Motor evoked potentials. Semin Anesth.

[37] Van Oostrom H, Doornenbal A, Schot A, Stienen PJ, Hellebrekers LJ (2011). Neurophysiological assessment of the sedative and analgesic effects of a constant rate infusion of dexmedetomidine in the dog. Vet J.

[38] Van Ham LM, Nijs J, Mattheeuws DR, Vanderstraeten GG (1996). Sufentanil and nitrous oxide anaesthesia for the recording of transcranial magnetic motor evoked potentials in dogs. Vet Rec.

[39] Van Ham LM, Nijs J, Vanderstraeten GG, Mattheeuws DR (1996). Comparison of two techniques of narcotic-induced anesthesia for use during recording of magnetic motor evoked potentials in dogs. Am J Vet Res.

[40] Vanhaesebrouck AE, Bhatti SF, Polis IE, Plessas IN, Van Ham LM (2011). Neuromyotonia in a Dachshund with clinical and electrophysiological signs of spinocerebellar ataxia. J Small Anim Pract.

[41] Poma R, Parent JM, Holmberg DL, Partlow GD, Monteit G, Sylvestre AM (2002). Correlation between severity of clinical signs and motor evoked potentials after transcranial magnetic stimulation in large-breed dogs with cervical spinal cord disease. J Am Vet Med Assoc.

[42] De Decker S, Van Soens I, Duchateau L, Gielen IM, Van Bree HJ, Binst DH (2011). Transcranial magnetic stimulation in Doberman Pinschers with clinically relevant and clinically irrelevant spinal cord compression on magnetic resonance imaging. J Am Vet Med Assoc.

[43] Van Soens I, Van Ham LM (2011). Assessment of motor pathways by magnetic stimulation in human and veterinary medicine. Vet J.

[44] Lall RR, Lall RR, Hauptman JS, Munoz C, Cybulski GR, Koski T (2012). Intraoperative neurophysiological monitoring in spine surgery: indications, efficacy, and role of the preoperative checklist. Neurosurg Focus.

